# The context of COVID-19 affected the long-term sleep quality of older adults more than SARS-CoV-2 infection

**DOI:** 10.3389/fpsyt.2024.1305945

**Published:** 2024-02-05

**Authors:** Vanessa Giffoni M. N. P. Peixoto, Lucas Alves Facci, Thiago C. S. Barbalho, Raíssa Nascimento Souza, Alice Mendes Duarte, Katie Moraes Almondes

**Affiliations:** ^1^ Post-graduation Program in Psychobiology, Universidade Federal do Rio Grande do Norte, Natal, Brazil; ^2^ Department of Clinical Medicine, Universidade Federal do Rio Grande do Norte, Natal, Brazil; ^3^ Department of Clinical Medicine Universidade Federal do Rio Grande do Norte, Natal, Brazil; ^4^ Department of Psychology, Universidade Federal do Rio Grande do Norte, Natal, Brazil

**Keywords:** sleep quality, older adults, COVID-19, SARS-CoV-2, long-term, post-COVID syndrome

## Abstract

**Introduction:**

Sleep problems are one of the most persistent symptoms of post-COVID syndrome in adults. However, most recent research on sleep quality has relied on the impact of the pandemic, with scarcely any data for older adults on the long-term consequences of COVID infection. This study aims to understand whether older individuals present persistently impaired sleep quality after COVID-19 infection and possible moderators for this outcome.

**Methods:**

This is a cross-sectional analysis of a longitudinal cohort study with 70 elders with 6-month-previous SARS-CoV-2 infection and 153 controls. The Pittsburgh Sleep Quality Index (PSQI) was used to assess sleep quality; Geriatric Depression Scale and Geriatric Anxiety Inventory for screening depression and anxiety. Demographics and comorbid conditions were collected.

**Results:**

The mean age of participants was 66,97 ± 4,64 years. There were no statistical differences in depression and anxiety between groups. Poor sleep quality was found in 52,9% and 43,8% of the COVID and control groups (*p*=.208). After controlling for multiple variables, all the following factors resulted in greater chances of poor sleep quality: female gender (OR, 2.12; *p*=.027), memory complaints (OR, 2.49; *p*=.074), insomnia (OR, 3.66; *p*=.032), anxiety (OR, 5.46; *p*<.001), depression (OR, 7.26; *p*=.001), joint disease (OR, 1.80; *p*=.050), glucose intolerance (OR, 2.20; *p*=.045), psychoactive drugs (OR, 8.36; *p*<.001), diuretics (OR, 2.46; *p*=.034), and polypharmacy (OR, 2.84; *p*=.016).

**Conclusion:**

Psychosocial burden in the context of the COVID-19 pandemic and pre-existing conditions seems to influence the sleep quality of older adults more than SARS-CoV-2 infection.

## 1 Introduction

Although the COVID-19 pandemic was declared over in May 2023 (1), it remains the most devastating disease in recent history, with unprecedented global spread leading to social and economic crises worldwide. For over two years, strict policy responses, including social distancing interventions, were adopted to prevent COVID transmission and protect the most vulnerable. Older adults were considered highly susceptible to COVID-19 complications and have consequently suffered more severe and prolonged social isolation (2), leading to deleterious impacts on their mental and physical health (3). On the other hand, some studies have pointed toward better adaptation and lower psychological distress in this age group compared to younger individuals (4, 5), possibly reflecting higher levels of resilience mediating COVID-19 stressors (5, 6).

Sleep has restorative and regulatory functions (7), being crucial to memory and other neurocognitive functions (8), hormone production (9), support of the immune system (10), metabolic control (7), and cardiovascular protection (11). Indeed, sufficient evidence links poor sleep health to a variety of adverse outcomes (12, 13). As a result, the American Heart Association has recently updated its ‘Life Essential’ guidelines to include sleep health as the newest crucial factor in optimizing cardiovascular health (14). Meanwhile, aging is associated with physiological changes in sleep architecture, circadian rhythm shifts, chronic medical and psychiatric illnesses, and polypharmacy (9, 15, 16), all concurring for a higher prevalence of sleep disorders (16, 17). As such, pre-existing sleep problems have been hypothesized to partially explain the unfavorable outcomes in the older age group during COVID infection (18).

Adequate sleep depends on various behavioral factors, including sunlight exposure, outdoor physical activities, sleeping schedules, nocturnal use of digital gadgets, and psychosocial issues (15, 19). Economic uncertainties, fear of getting infected, and loneliness are examples of the psychological distress that arose from the COVID-19 outbreak, not to mention social isolation and home confinement, which intensely disrupted daily routines, daylight exposure, and circadian rhythms (18, 20).

Given these facts, most recent research on sleep quality has addressed the impact of the pandemic itself on the quality of sleep of the overall population (19, 21, 22). After the first year of the COVID-19 pandemic, two systematic reviews and meta-analyses reported pooled prevalence of sleep problems between 18% to 37,5% in the general population, contrasting with 57% to 74,8% among patients who recently recovered from infection (19, 23), therefore suggesting that SARS-CoV-2 infection might implicate future sleep problems. With a look at post-COVID syndrome, Chen et al. (24) reported the prevalence of sleep problems of 11% (95% CI, 0.05–0.23) up to 120 days post-infection in a meta-analysis that gathered information from 1,680,003 COVID‐19 patients. Interestingly, Huang et al. (25) followed up on 1,119 adult Chinese patients (median age of 57 years) 2 years after recovering from SARS‐CoV‐2 infection and found at least one persistent symptom in as much as 55% of patients, fatigue and sleep disturbances being the most common complaints. Remarkably, older age was one of the strongest predictors of symptom persistence in this cohort. Nonetheless, concerning older patients, few studies have specifically investigated whether sleep might be persistently disturbed long after COVID infection (26). Hence, this study aims to understand whether older individuals have long-term impacts on sleep quality after COVID-19 infection and which factors may influence this outcome.

## 2 Methods

### 2.1 Study design and population

This is a cross-sectional analysis of a longitudinal cohort study with a total follow-up of two years, comprising two groups of older adults, either previously infected by the novel coronavirus or healthy peers. Recruitment took place in March 2021, during the second wave of the pandemic, in which the Gamma variant was circulating. Eligible participants were those aged 60 to 80 with a formal education of over four years. COVID (+) participants were recruited from a private laboratory in Natal, Brazil, among all 527 patients in this age group diagnosed with SARS-CoV-2 infection by RT-PCR technique this month. Healthy controls, which means those who had never been infected by SARS-CoV-2, were engaged through social media announcements, in whom the serologic status was tested through the ELISA technique to rule out previously unknown disease. Of note, these subjects had not received any COVID vaccine yet. Individuals with previous cognitive decline, uncontrolled psychiatric conditions, recent stroke, heart attack, or cardiac arrest were excluded. The general inclusion and exclusion criteria were applied to both cohorts. After inclusion and exclusion criteria were applied and due to the longitudinal nature of the study and the anticipated proportion of controls to contract COVID-19 during the follow-up, a final of 70 COVID and 153 control participants comprised the initial sample. They were submitted to three comprehensive in-person interviews by a certified geriatrician, neuropsychologist, and trained research assistants. The data presented in this paper was collected between May and September 2021 and relates to the first of three assessments of the prospective follow-up. In the COVID group, six months elapsed from the COVID-19 diagnosis to the assessment.

### 2.2 Instruments

A structured questionnaire was used to record data on demographics and self-report of comorbid conditions. Depression was screened by the Geriatric Depression Scale (GDS-15), ranging from 0 to 15, with higher scores indicating higher depression probability (27). Anxiety was suggested by scores higher than 10 on the Geriatric Anxiety Inventory (GAI) (28). The Pittsburgh Sleep Quality Index (PSQI) (29, 30) was used to assess sleep quality within the last month. It is a 19-item questionnaire addressing seven components of sleep quality: subjective sleep quality, sleep latency, sleep duration, sleep efficiency, sleep disturbance, sleep medication intake, and daytime dysfunction. Each component score is rated on a 0–3 scale, where 3 on the Likert scale corresponds to a negatively extreme response. The total score leads to a sum of up to 21 points, in which PSQI scores > 5 indicate poor sleep quality and ≤ 5 indicate good sleep quality.

### 2.3 Statistics

Statistical analyses were performed with the IBM^®^ Statistical Package for Social Sciences (SPSS) version 23. Descriptive and inferential analyses were completed for demographic and clinical data, GDS-15, GAI, and PSQI. According to the Shapiro-Wilk test, normally distributed continuous variables were analyzed by Student’s t-test or Mann–Whitney test for non-normally distributed variables. Pearson’s chi-square or Fisher’s tests were used for group comparisons on nominal variables. A *p*-value <.05 was considered statistically significant. All categorical variables with *p*-values <.20 were tested through a multivariate logistic regression, identifying poor sleep quality predictors.

### 2.4 Ethics

The study was conducted by ethical guidelines after approval by the local Research Ethics Committee of UFRN – Campus Central, Natal, Brazil, under process number 44011221.8.0000.5537. Written informed consent was collected from all participants before conducting the study. All information was kept confidential.

## 3 Results

### 3.1 Sociodemographic and clinical characteristics

A total of 223 older adults aged 60 to 78 participated in the study (70 with previous exposure to SARS-CoV-2 - COVID (+) and 153 healthy controls). The mean age of participants was 66,97 ± 4,64 years. The female gender accounted for approximately 70% of participants. Other sociodemographic characteristics of the sample are summarized in **Table 1**. Frequencies of the leading clinical conditions and the medications in use are summarized in **Table 2**. Most participants reported exercising regularly (**Table 1**). Depression estimate, according to the GDS-15 was approximately 8% in both groups; anxiety measured by GAI was 17.1% and 11.1% in the COVID and control groups, respectively. There were no statistically significant differences in depression and anxiety between groups (**Table 3**).

**Table 1:** Comparison of sociodemographic and clinical characteristics between COVID and CONTROL groups during the second wave of COVID-19.

Variables	COVID (n = 70)	CONTROL (n = 153)	Statistics	*p* value
Age - M ± DP 60 – 69 - n (%) 70 – 78 - n (%)	67.11 ± 4.95 46 (65.7) 24 (34.3)	66.90 ± 4.51 105 (68.6) 48 (31.4)	0.958^(1)^ 0.186^(2)^	0.813 0.666
Gender - n (%) Female Male	46 (65.7) 24 (34.3)	109 (71.2) 44 (28.8)	0.692^(2)^	0.405
Marital status - n (%) Single Married Divorced Widower	01 (1.5) 50 (71.4) 8 (11.4) 11 (15.7)	14 (9.2) 89 (58.2) 27 (17.6) 23 (15)	5.894^(3)^	0.052
Schooling - n (%) 4 – 8 years 9 years 12 years Higher level Postgraduate	2 (2.9) 6 (8.6) 25 (35.7) 25 (35.7) 12 (17.1)	5 (3.3) 6 (3.9) 33 (21.6) 68 (44.4) 41 (26.8)	7.578^(3)^	**0.000**
Income - n (%) BRL < 5.000 BRL 5.000 e 10.000 BRL 10.000 e 23.000 Over BRL 23.000	22 (31.43) 28 (40) 17 (24.3) 3 (4.3)	44 (28.76) 37 (24.2) 61 (39.9) 11 (7.1)	9.815^(3)^	**0.042**
Occupation - n (%) Retired Working Unemployed	49 (70) 21 (30) 0	107 (69.9) 38 (24.8) 8 (5.2)	0.716^(3)^	0.397
Complications of acute illness - n (%) Pneumonia In-hospital care Use of supplemental O_2_	38 (54.3) 16 (22.9) 16 (22.9)	- - -		
Exercise practice - n (%)	36 (51.4)	95 (62.1)	1.934^(2)^	0.164
Memory complaints - n (%)	40 (57.1)	65 (42.5)	3.574^(2)^	**0.029**
Sleep problems ^¶^ - n (%) Insomnia Sleep apnea Nightmares Daily napping	36 (51.4) 32 (45.7) ^§^ 08 (11.4) ^§^ 03 (4.3) ^§^ 49 (70.0) ^§^	59 (38.6) 52 (34.0) ^§^ 12 (7.8) ^§^ 01 (0.7) ^§^ 95 (62.1) ^§^	3.251^(2)^ 2.813^(2)^ 0.756^(2)^ 3.579^(2)^ 1.313^(2)^	0.071 0.093 0.450 0.093 0.252
Sleep complaint time ^¶^ - n (%) More than 1 year ago Less than 1 year ago No problem sleep	26 (37.1) 10 (14.3) 34 (48.6)	53 (34.6) 06 (3.9) 94 (61.4)	8.660^(2)^	**0.013**

Values expressed as mean (M) ± standard deviation (SD) or number of subjects (n)/frequencies (%). (1) Mann-Whitney (U) test for comparison between the medians of the groups; (2) Chi-square test (X2) and (3) Fisher test for differences between groups. (^¶^) Characteristics of self-reported sleep problems. (^§^) Frequency in relation to the total population of each group. Significant results (p < 0.05) in bold. COVID, individuals with previous COVID-19; BRL, (Brazilian currency).

**Table 2:** Comparison of the prevalence of self-reported comorbidities and medications in use between the COVID (at the time of collection) and CONTROL groups.

	COVID (n = 70) n (%)	CONTROL (n = 153) n (%)	Statistics	*p* value
Comorbidities				
Dyslipidemia	44 (62.9)	82 (53.6)	1.676^(2)^	0.195
Hypertension	35 (50.0)	87 (56.9)	0.913^(2)^	0.339
Joint diseases	25 (35.7)	67 (43.8)	1.293^(2)^	0.256
Diabetes	17 (24.3)	33 (21.6)	0.204^(2)^	0.652
Overweight	13 (18.6)	26 (17.0)	0.083^(2)^	0.773
Anxiety	13 (18.6)	34 (22.2)	0.385^(2)^	0.535
Hypothyroidism	13 (18.6)	20 (13.1)	1.152^(2)^	0.283
Osteoporosis	13 (18.6)	35 (22.9)	0.527^(2)^	0.468
Hystory of cancer	12 (17.1)	20 (13.1)	0.648^(2)^	0.421
Glucose intolerance	11 (15.7)	29 (19.0)	0.343^(2)^	0.558
Arrhythmia	10 (14.3)	20 (13.1)	0.061^(2)^	0.805
Coronary disease	09 (12.9)	14 (9.2)	0.713^(2)^	0.398
Depression	09 (12.9)	18 (11.8)	0.054^(2)^	0.816
Number of comorbidities 0 or 1 comorbidity 2 or more comorbidities	10 (14.3) 60 (85.7)	23 (15.0) 130 (85.0)	0.021^(2)^	0.884
Medications				
Statins	42 (60.0)	69 (45.1)	4.266^(2)^	**0.039**
ACEi/ARB	27 (38.6)	64 (41.8)	0.211^(2)^	0.646
Vitamin D	22 (31.4)	63 (41.2)	1.935^(2)^	0.164
Oral antidiabetics	20 (28.6)	52 (34.0)	0.644^(2)^	0.422
Antidepressant	18 (25.7)	29 (19.0)	1.319^(2)^	0.251
Betablockers	18 (25.7)	37 (24.2)	0.061^(2)^	0.806
Platelet antiaggregants	15 (21.4)	17 (11.1)	4.160^(2)^	**0.041**
Thyroid hormone	14 (20.0)	23 (15.0)	0.856^(2)^	0.355
Benzodiazepines	09 (12.9)	23 (15.0)	0.185^(2)^	0.667
Diuretics	09 (12.9)	27 (17.6)	0.814^(2)^	0.367
Proton pump inhibitors	08 (11.4)	16 (10.5)	0.047^(2)^	0.828
Number of medications 0 to 4 medications 5 or more medications	52 (74.3) 18 (25.7)	127 (83.0) 26 (17.0)	2.306^(2)^	0.129

Values expressed as the number of individuals (n)/frequencies (%). Comorbidities listed with prevalence >5% in descending order of frequency according to COVID group. (2) Chi-square (X2) test for differences between groups. Significant results (p < 0.05) in bold. COVID, individuals with previous COVID-19; ACEi, angiotensin-converting enzyme inhibitors; ARB, angiotensin II receptor blockers.

**Table 3:** Comparison of the results of screening tests for poor sleep quality, depression, and anxiety (PSQI/GDS-15/GAI) between the COVID group (at the time of collection) and the control group (absolute values and respective binary classification according to cut-off points when available).

	COVID (n = 70)	CONTROL (n = 153)	Statistics	*p* value
PSQI - M ± SD Altered - n (%) Subjective sleep quality Sleep latency Sleep duration Habitual sleep efficiency Sleep disturbance Use of sleep medication Daytime dysfunction	6.26 ± 3.45 37 (52.9) 1.14 ± 0.73 1.39 ± 1.22 0.91 ± 0.96 0.43 ± 0.81 1.21 ± 0.51 0.67 ± 1.19 0.51 ± 0.68	5.88 ± 3.49 67 (43.8) 0.97 ± 0.65 1.19 ± 1.09 1.08 ± 0.94 0.33 ± 0.68 1.22 ± 0.46 0.5 ± 1.08 0.59 ± 0.71	4928.5^(1)^ 1.586^(2)^ 4660^(1)^ 4928^(1)^ 5925^(1)^ 5145^(1)^ 5409^(1)^ 4942^(1)^ 5656^(1)^	0.338 0.208 0.083 0.320 0.179 0.535 0.874 0.197 0.450
GDS-15 - M ± SD Altered - n (%)	1.97 ± 2.25 06 (8.6)	2.03 ± 2.1 12 (7.8)	5561^(1)^ 0.034^(2)^	0.638 0.853
GAI - M ± SD Altered - n (%)	4.06 ± 4.49 12 (17.1)	3.59 ± 4.19 17 (11.1)	5149^(1)^ 1.544^(2)^	0.640 0.214

Values expressed as mean (M) ± standard deviation (SD) or number of subjects (n)/frequencies (%). (1) Mann-Whitney (U) test for comparison between medians; (2) Chi-square (x2) test for differences between groups. COVID. individuals with previous COVID-19; TICS-m, Telephone interview for cognitive status assessment - modified; MoCA-B, Montreal Cognitive Assessment - Basic; GDS-15, Geriatric depression scale - 15-item version; GAI, Geriatric anxiety inventory; IQSP, Pittsburgh sleep quality index.

### 3.2 Sleep analysis

Participants from the COVID group reported more sleep difficulties (51.4%) compared to the control group (38.6%), although this difference was not statistically significant (X2 = 3.251, gl=1, *p*= .071). Insomnia and sleep apnea were the most cited sleep problems during the second wave of the pandemic. Regarding individuals who reported sleep-related conditions, 72% of the COVID group and 90% of the control group already presented this complaint for over a year. Daily naps were reported by 70% of the COVID group and 62.1% of the control group (**Table 1**). Mean PSQI scores were 6,26 ± 3,45 in the COVID group and 5,88 ± 3,49 in the control group (U=4928,5; *p*= .338). According to a cut-off point of 5 in the PSQI, 52,9% and 43,8% of the COVID and control groups had poor sleep quality (X^2 = ^1,586, df= 1, *p*= .208 - **Table 3**).

After controlling for multiple variables, logistic regression analysis identified some factors associated with greater chances of having poor quality of sleep according to the PSQI scale: female gender (OR, 2.12; 95% CI, 1.09–4.11; *p*= .027), individuals with memory complaints (OR, 2.49; 95% CI, 0.92–6.76; *p*= .074), insomnia (OR, 3.66; 95% CI, 1.12–11.96, *p*= .032), self-reported anxiety (OR, 5.46; 95% CI, 2.46–12.11; *p*<.001), self-reported depression (OR, 7.26; 95% CI, 2.30–22.88, *p*= .001), joint disease (OR, 1.80; 95% CI, 0.99–3.26; *p*= .050), and glucose intolerance (OR, 2.20; 95% CI, 1.02–4.76; *p*= .045). Also, those in use of psychoactive drugs (OR, 8.36; 95% CI, 4.14–16.87; *p*<.001), diuretics (OR, 2.46; 95% CI, 1.07–5.66; *p*= .034), and polypharmacy (OR, 2.84; 95% CI, 1.22–6.63; *p*= .016) were more likely to have poor sleep quality (**Table 4**).

**Table 4:** Odds Ratio for poor sleep quality (PSQI >5).

Variables	OR (95% CI)	*p* value
Female gender Insomnia Subjective memory complaints Anxiety (self-reported) Depression (self-reported) Joint diseases Glucose intolerance Psychoactive drugs Diuretics Polypharmacy	2.12 (1.09 – 4.11) 3.66 (1.12 – 11.96) 2.49 (0.92 – 6.76) 5.46 (2.46 – 12.11) 7.26 (2.30 – 22.88) 1.80 (0.99 – 3.26) 2.20 (1.02 – 4.76) 8.36 (4.14 – 16.87) 2.46 (1.07 – 5.66) 2.84 (1.22 – 6.63)	**0.027** **0.032** 0.074 **<0.001** **0.001** **0.050** **0.045** **<0.001** **0.034** **0.016**

Values expressed as absolute number. Significant results (p <.05) in bold.

## 4 Discussion

Sleep problems seem to be one of the most persistent symptoms of post-COVID syndrome in adults older than 18 years, with a variety of predisposing factors, such as older age, female gender, the severity of the acute illness, the time of follow-up from recovery, and premorbid conditions (24, 25). Our study demonstrated a high proportion of sleep complaints among older adults during the second wave of the pandemic, regardless of previous SARS-CoV-2 infection. Nearly half of the participants reported poor sleep quality according to PSQI. Although PSQI mean score and its ‘subjective sleep quality’, ‘latency’ and ‘efficiency’ components were higher in the COVID group, no statistically significant difference was found when comparing them to healthy controls (**Table 3**).

Contrary to what we expected, previous SARS-CoV-2 infection did not influence sleep quality in persons 60 and over six months after infection. We have hypothesized some explanations for this finding: (1) the older age group has been assumed to be the most at-risk population for COVID-19, and most of them were submitted to more prolonged and strict social isolation. As so, the psychosocial burden and confinement measures due to the COVID-19 pandemic *per se* might have been the primary responsible for sleep problems in both groups, including that of healthy controls (19, 21); (2) it is intuitive that the severity of SARS-CoV-2 infection possibly implicates further neurological sequelae (24, 31). In our study, only one in every four patients of the COVID (+) group needed in-hospital care (**Table 1**), leading us to extrapolate that most of this cohort experienced mild illness, resulting in less probable post-COVID symptoms; (3) there is scarcely any data on the Gamma variant post-COVID syndrome, and it is plausible that this strain causes less disturbed sleep like the Delta variant (32); (4) higher formal schooling (33) and income (34) have been associated with better-coping strategies (24) and pointed as protective factors against worsening sleep quality. Surprisingly, both cohorts in this study have been mainly composed of subjects with high income and at least a higher degree of education, which could have prevented participants from experiencing worse sleep complaints; (5) finally, as mentioned elsewhere in this article, the aging process is associated with greater resilience to stressful events, possibly resulting in less deleterious impacts on the sleep when compared to younger individuals (5, 6).

After controlling for multiple variables, our results indicated greater chances of inadequate sleep in women, people with self-reported anxiety and depression, arthritis, glucose intolerance, cognitive complaints, psychoactive drugs, diuretics, and polypharmacy. Although men are more susceptible to severe forms of acute COVID-19, women seem to be disproportionally affected by long COVID symptoms (35, 36), also in sleep issues (37). Biological and psychosocial factors might explain such differences, starting with women being more likely to report somatic symptoms (38). Additionally, traditional social roles in low and middle-income countries, such as caring for family members and domestic duties, might have overwhelmed women during COVID-19 pandemic and implicated greater stress levels and poorer sleep quality (39). Biological sex differences in structure and hormones also justify discrepancies in sleep architecture and disorders (see Mallampalli and Carter for detailed review) (40).

Importantly, participants suffering from depression and anxiety experienced chances of poor sleep 5 to 7 times higher, in line with the entire body literature on the reciprocal relationship between sleep disturbances and psychiatric comorbidities (19, 41–43). In keeping with this view, taking psychoactive drugs also hugely impacted on worse sleep quality, probably through different mechanisms. First, psychoactive compounds potentialize monoaminergic transmission and receptors’ activity, affecting sleep integrity (44); second, some antidepressants may exacerbate primary sleep disturbances such as restless legs syndrome and periodic limb movement, whereas benzodiazepines and opioids may accentuate obstructive sleep apnea (44); finally, the relationship between psychoactive drugs and sleep quality might indirectly reflect the link between pre-existing neuropsychiatric conditions and sleep issues. However, our study has neither identified a single pharmacological agent underlying this association nor explored whether these drugs may have been prescribed as sleeping aids.

Diuretic drugs also doubled the chances of poor sleep in our sample of elders. Nocturia was the main reason for disturbed sleep in a sample of 1424 older individuals (45) and implicates reduced quality of life, diurnal somnolence, and falls during awakenings (46). Polypharmacy - the regular use of at least five drugs - also increased the chances of bad sleep quality in our sample, as was described in previous studies (47, 48). Conversely, a study conducted with elders living in care facilities in Malaysia found no statistical relationship between polypharmacy and sleep quality, although individuals with poor sleep used more drugs compared to those with good sleep (49). In the last few decades, polypharmacy has been on the rise among the geriatric population, with current estimates varying from 26,3 to 39,8% across European countries and Israel (50). The risk of potential drug-drug interactions increases dramatically with the number of drugs used, as do drug-disease interactions, resulting in unfavorable outcomes including falls, hospitalization, a decline in physical function, frailty, more sleep problems, and mortality (51).

Nonetheless, it is hard to disentangle the side effects of multiple drugs from the underlying comorbidities for which the drugs were prescribed, including in the context of sleep (15). This was particularly relevant in our sample, as people with chronic conditions like arthritis and glucose intolerance also exhibited greater chances of disturbed sleep. Much is known regarding the somewhat reciprocal association between sleep disturbance and chronic pain, in which psychological factors play an essential role (52). Osteoarthritis-related pain has also been previously associated with decreased sleep quality (53). Disturbed sleep has been recognized as a risk factor for metabolic and cardiovascular diseases (54); vice versa, sleep duration and quality directly impact glycemic control (55).

Surprisingly though, patients with subjective cognitive complaints had twice higher chances of poor sleep; however, given the transversal nature of our preliminary analysis, it is possible that the natural direction of this relationship might be inverse. Links between sleep and cognitive decline have been increasingly debated in the last few years (56, 57) and seem to be bidirectional (56). Sleep disturbances might be implicated in higher risks of subjective cognitive decline (57, 58), mild cognitive impairment (MCI), and Alzheimer’s disease (AD) (59, 60). On the other hand, sleep disturbances frequently co-occur in neurodegenerative disorders from the earliest through late stages, as seen, for instance, in AD, in which patients may present sleep fragmentation, shorter duration and changes in architecture, diurnal somnolence and sleep-wake cycle inversion (61). In some neurodegenerative conditions, such derangements start in preclinical stages, as observed in REM sleep behavior disorder and synucleinopathies (62).

An important strength of this study resides in the older age target. In times of social restriction measures, we could conduct personal interviews with a significant number of senior volunteers and follow up with them using validated assessment scales to evaluate sleep quality and psychiatric morbidity. Soon, the longitudinal nature of our data will help to add crucial information about the long-term effects of SARS-CoV-2 infection on the sleep quality of older people. Having healthy controls with no previous COVID-19 infection (as verified through serological exams) is another strength of our work, as well as recruiting volunteers and collecting data before COVID vaccination. Finally, researchers were blind to the previous exposure of participants to SARS-CoV-2, which helped to reduce bias. However, we recognize some limitations of this research. First, since it covers people aged 60 and over, the results may not be generalizable to younger populations. Second, participants’ high educational level and income possibly reflected their more significant interest in research volunteering and may represent a selection bias. In this perspective, education might have acted as a protective factor toward better sleep quality, as discussed above. Third, participants, not medical records, informed clinical data concerning comorbidities, medications, and COVID infection. Fourth, regarding psychiatric morbidity, data was either extracted from participants’ self-report on their comorbidities or through validated questionnaires (GDS-15 and GAI). It is possible that under the high prevalence of psychoactive compounds in our sample, current depressive and anxious symptoms were somewhat controlled, and the screening questionnaires were less effective than the self-report of pre-existing depression and anxiety. Finally, although PSQI is a validated and widespread instrument to assess sleep quality, participants were not submitted to polysomnography to investigate detailed sleep parameters.

## 5 Conclusion

Psychosocial burden in the context of the COVID-19 pandemic and pre-existing illness seems to have influenced the sleep quality of older adults more than the Gamma variant of SARS-CoV-2 infection *per se*. It is crucial to stress the multifactorial interference of diseases and medications in the sleep of older adults. Healthcare providers should be aware of potential drug-drug and drug-disease interactions and specific drugs affecting sleep. Our longitudinal data will help clarify further aspects concerning the trajectory of SARS-CoV-2 infection recovery, covering sleep quality.

## Data availability statement

The raw data supporting the conclusions of this article will be made available by the authors, without undue reservation.

## Ethics statement

The studies involving humans were approved by Comitê de Ética em Pesquisa (Campus Central) UFRN. The studies were conducted in accordance with the local legislation and institutional requirements. The participants provided their written informed consent to participate in this study.

## Author contributions

VP: Conceptualization, Data curation, Formal Analysis, Investigation, Methodology, Project administration, Supervision, Visualization, Writing – original draft, Writing – review & editing. LF: Data curation, Investigation, Resources, Writing – original draft. TB: Data curation, Investigation, Resources, Writing – original draft. RS: Data curation, Investigation, Resources, Writing – original draft. AD: Data curation, Investigation, Resources, Writing – original draft. KA: Conceptualization, Funding acquisition, Methodology, Project administration, Resources, Supervision, Visualization, Writing – review & editing.
